# Effects of Three-Dimensional Calcium Chloride-Crosslinked Alginate–Gelatin Hydrogels on Osteo-Odontogenic Differentiation of Odontoblast-like Cells

**DOI:** 10.3390/polym18091024

**Published:** 2026-04-23

**Authors:** Taufik Abdullah Mappa, Hung-Yang Lin, Hsieh-Tsung Shen, Keng-Liang Ou, Yu-Sin Jennifer Ou, Chi-Hsun Tsai, Takashi Saito, Yung-Kang Shen

**Affiliations:** 1 School of Dentistry, College of Oral Medicine, Taipei Medical University, Taipei 110, Taiwan; d204111004@tmu.edu.tw; 2 Division of Clinical Cariology and Endodontology, Department of Oral Rehabilitation, School of Dentistry, Health Sciences University of Hokkaido, Tobetsu 061-0293, Hokkaido, Japan; ethanshen@eg-bio.com (H.-T.S.); lilaln0302@hoku-iryo-u.ac.jp (C.-H.T.); 3 Department of Oral and Maxillofacial Surgery, Faculty of Dentistry, Hasanuddin University, Makassar 90245, Indonesia; 4 Department of Dentistry, Fu Jen Catholic University Hospital, Fu Jen Catholic University, New Taipei City 242, Taiwan; a00207@mail.fjuh.fju.edu.tw; 5 Yu Ding Global Cancer Research Foundation, Taipei 115, Taiwan; 6 EG BioMed US Inc., Covina, CA 91722, USA; 7 Ji Yan Biomedical Co., Ltd., Taipei 115, Taiwan; 8 Department of Dentistry, Taipei Medical University, Shuang Ho Hospital, New Taipei City 235, Taiwan; klouyu@gmail.com; 9 3D Global Biotech Inc. (Spin-Off Company from Taipei Medical University), New Taipei City 221, Taiwan; 10 General Biology, Warren College, University of California, San Diego, CA 92093, USA; y3ou@ucsd.edu; 11 School of Dental Technology, College of Oral Medicine, Taipei Medical University, Taipei 110, Taiwan

**Keywords:** alginate-gelatin hydrogels, calcium chloride, crosslinking, osteo-odontogenic differentiation, dentin regeneration, odontoblast-like cells

## Abstract

This study evaluated whether three-dimensional alginate–gelatin hydrogels (AGHs) crosslinked with calcium chloride (CaCl_2_) enhance the osteo-odontogenic differentiation of odontoblast-like cells in vitro. Two seeding configurations were compared: inter-hydrogel (INT) surface seeding and intra-hydrogel (INTR) encapsulation. Here, the MDPC-23 cells were cultured in AGHs crosslinked with 70 or 100 mM CaCl_2_ and assessed for proliferation, cytoskeletal morphology, alkaline phosphatase (ALPase) activity, osteo-odontogenic gene expression, and mineralized nodule formation. After 7 days, cell proliferation was significantly greater in the alginate–gelatin hydrogel (AGH) groups than in the control group. Cells in the intra alginate–gelatin hydrogel 100 (INTR-AGH100) remained predominantly rounded, whereas those in the inter alginate–gelatin hydrogel 100 (INT-AGH100) formed irregular clusters on the hydrogel surface. ALPase activity was highest in INTR-AGH100 at the early stage of culture. Both INT-AGH100 and INTR-AGH100 showed significantly increased expression of DSPP, DMP-1, BSP, OCN, OPN, and Runx-2, together with enhanced mineralized nodule formation. Although no significant differences were detected between the two seeding strategies in all assays, distinct morphological patterns were observed, and the INTR configuration showed relatively greater early differentiation-related activity. These findings suggest that 100 mM CaCl_2_-crosslinked AGHs provide a favorable three-dimensional microenvironment under the present experimental conditions and represent a promising in vitro scaffold platform to support future studies of scaffold-guided dentin regeneration.

## 1. Introduction

Regenerative dentistry has advanced considerably in recent years, particularly in strategies aimed at preserving the vitality of dentin-pulp tissue. These advances rely on tissue engineering approaches that integrate cells, bioactive factors, and hydrogel scaffolds to stimulate functional tissue regeneration and to provide alternatives to conventional vital pulp therapy materials [1,2,3,4,5]. In this context, hydrogel-based constructs have attracted increasing attention because they can support reparative dentin formation, provide a protective mineralized barrier over exposed pulp, and help maintain the structure and function of the pulp tissue [1,2,4,5,6,7,8,9,10].

Compared with the natural process of the development of dentin–pulp complex, current regenerative approaches rely on biomaterials that create a permissive microenvironment for cellular activity rather than directly reproducing all aspects of native tissue formation [4,5,11,12,13]. The selection of an appropriate scaffold is therefore critical [14]. An ideal hydrogel scaffold should support cell survival, promote cell–matrix interactions, facilitate mineralization, and provide structural properties suitable for dentin-related tissue regeneration [15,16]. In particular, hydrogel-based carriers capable of delivering multipotent odontoblast-like cells may enhance osteo-odontogenic differentiation and thereby support hard-tissue formation [17,18,19].

Among candidate scaffold materials, alginate–gelatin hydrogels have been widely studied for tissue-engineering applications because of their favorable physicochemical and biological properties [20,21,22]. Alginate provides a porous hydrogel network that supports the diffusion of nutrients and signaling molecules, whereas gelatin contributes cell-interactive domains that facilitate adhesion and differentiation [17,20]. Together, these components can form biocompatible scaffolds with appropriate structural integrity and rheological behavior for cell delivery [14,21,22].

The alginate–gelatin system can be ionically crosslinked with calcium chloride, allowing a liquid-to-gel transition through physicochemical interactions between Ca^2+^ ions and alginate chains [23,24]. As a crosslinking agent, calcium chloride forms ionic bridges between polymer chains, thereby stabilizing the hydrogel network [25,26]. Depending on crosslinking conditions such as ion concentration, composition, and temperature, calcium chloride–crosslinked hydrogels may develop micro- to macro porous structures that influence cell attachment and matrix interactions [13,25,26,27]. These characteristics make calcium chloride–crosslinked alginate–gelatin hydrogels attractive for fabricating three-dimensional constructs for regenerative applications [23,28]. Previous studies have reported favorable biocompatibility, thermal stability, and cell-supporting properties in alginate–gelatin systems crosslinked with calcium chloride [20,27]. However, the effects of defined calcium chloride concentrations on alginate–gelatin hydrogels containing odontoblast-like cells remain insufficiently characterized.

Another important consideration is the spatial relationship between cells and the hydrogel scaffold. Odontoblast-like cells have been reported to behave differently in two-dimensional and three-dimensional culture environments [29]. Recent studies have therefore used two distinct hydrogel culture strategies: intra-hydrogel cell encapsulation and inter-hydrogel surface seeding [25,30]. The intra-hydrogel approach embeds cells within the hydrogel matrix, thereby providing a three-dimensional microenvironment that more closely resembles the in vivo extracellular milieu, although nutrient diffusion may be restricted in the inner region of the construct [27]. In contrast, the inter-hydrogel approach places cells on the hydrogel surface, allowing greater exposure to surface cues and facilitating early adhesion and spreading, but it may provide less support for three-dimensional differentiation [11,27]. A direct comparison of these two seeding strategies under defined calcium chloride crosslinking conditions may therefore provide useful insight into the design of hydrogel-based dentin-regeneration models [30]. Although the alginate–gelatin hydrogels have been somewhat effective, they are still limited by factors related to tissue regeneration assessment using odontoblast-like cells.

Accordingly, to drive the line forward and expand the optimal understanding of calcium chloride–crosslinked three-dimensional alginate–gelatin hydrogels promote the proliferation, mineralization, and osteo-odontogenic differentiation of odontoblast-like cells, and whether these responses differ between intra-hydrogel and inter-hydrogel seeding strategies.

## 2. Materials and Methods

### 2.1. Preparation of Alginate–Gelatin Hydrogels

Alginate–gelatin hydrogels (AGHs) were kindly provided by 3D Global Biotech Inc. (New Taipei City, Taiwan) and consisted of 4% *w*/*v* sodium alginate and 2% *w*/*v* gelatin. Calcium chloride (CaCl_2_, Nacalai Tesque Inc., Kyoto, Japan) solutions were preapred in 50 mL of distilled water (Invitrogen, Thermo Fisher Scientific Inc., Waltham, MA, USA) at concentrations of 70 mM and 100 mM. After cell seeding, the calcium chloride solutions were applied to the alginate–gelatin hydrogels to induce ionic crosslinking. Followed study from [21,22], the constructs were allowed to react for 5 min at room temperature under identical conditions across all groups. Residual CaCl_2_ solution was then removed, and the constructs were gently rinsed with phosphate-buffered saline (PBS; Gibco, Thermo Fisher, UK) before culture. Based on our previous study, 50 mM CaCl_2_ was used as the comparison control condition [21]. Preliminary observations showed that CaCl_2_ concentraions below 50 mM did not produced stable gelation of the alginate–gelatin mixture under the present experimental conditions.

### 2.2. Cell Proliferation and Morphological Observation

**Figure 1:**
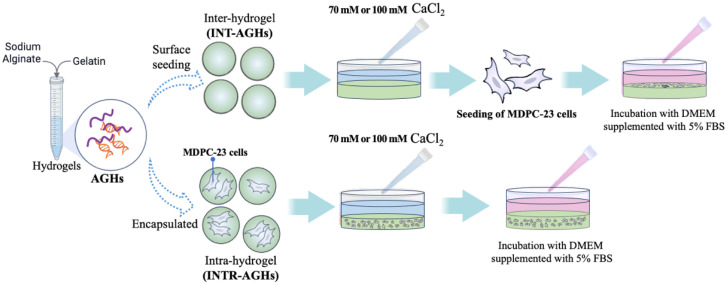
Schematic illustration of alginate–gelatin hydrogels preparation and MDPC-23 cell seeding using inter-hydrogel (INT-AGH) and intra-hydrogel (INTR-AGH) methods.

MDPC-23 was used as an odontoblast-like cell model. Cells were cultured in Dulbecco’s Modified Eagle’s Medium (DMEM; Sigma-Aldrich, St. Louis, MO, USA) supplemented with 5% fetal bovine serum (FBS, Thermo Fisher Scientific Inc., Waltham, MA, USA). For cell-related experiments, 100 µL of alginate–gelatin hydrogel was placed in each well of a 96-well plate. MDPC-23 cells were introduced at a density of 5 × 10^3^ cells/well using either the inter-hydrogel (INT-AGH; surface seeding) or intra-hydrogel (INTR-AGH; encapsulation) method (Figure 1). Subsequently, 100 µL of either 70 mM or 100 mM CaCl_2_ solution was added, and the constructs were allowed to crosslink for 5 min. Before culture, the constructs were gently rinsed twice with PBS (Gibco, Thermo Fisher, Paisley, UK). The cell-laden constructs were evaluated on days 3, 5, and 7 of culture using the Cell Counting Kit-8 assay (Dojindo, Rockville, MD, USA) to detect cell viability. Briefly, 10 μL/well of CCK-8 reagent was transferred and incubated at 37 °C for 1 h, followed added 50 μL/well of sodium citrate (Sigma-Aldrich, Darmstadt, Germany) to each well prior to analysis. A spectrophotometer (Varioskan LUX, Thermo Scientific, Waltham, MA, USA) was used to measure absorbance at 450 nm. All procedures were performed in triplicate, and cell morphology was observed under phase-contrast microscopy (Olympus, Tokyo, Japan).

### 2.3. Fluorescence Staining of Actin Cytoskeleton

Fluorescence staining was performed to visualize the cytoskeleton morphology. Briefly, 400 μL of alginate–gelatin hydrogels with 4 × 10^4^ cells/well was placed in 12-well microplates. The constructs were then treated with 400 μL of either 70 mM or 100 mM CaCl_2_, react for 5 min. After removal of the culture medium, the cells were gently rinsed with phosphate-buffered saline (PBS, Gibco, UK) and fixed in 10% neutral buffer formalin (Wako, Osaka, Japan) for 10 min. The cytoskeleton was visualized using ActinRed^TM^ 555 ReadyProbes^TM^ reagent (Invitrogen, Waltham, MA, USA) after permeabilized with 0.1% Triton X-100 (Sigma-Aldrich, USA). Continue to incubate for 30 min, then specimens were gently rinsed with PBS and counterstained with NucBlue^TM^ reagent (Invitrogen). All reactions was carried out in the dark state. Cell morphology was visualized on days 3, 5, and 7 using an inverted fluorescent microscope (Nikon ECLIPSE Ti 2 series, Tokyo, Japan).

### 2.4. ALPase Activity

For ALPase analysis, 2 × 10^4^ of MDPC-23 cells/well were seeded into 24-well microplates containing 300 μL of AGHs. The constructs were then treated with 300 μL of either 70 mM or 100 mM CaCl_2_, react for 5 min and gently rinsed twice with PBS (Gibco, Thermo Fisher) before incubation. The constructs were cultured for 5 or 7 days in regular medium consisting of DMEM supplemented with 5% FBS. To evaluate osteo-odontogenic differentiation, induction medium containing DMEM, 5% FBS, β-glycerophosphate (β-GP, Wako, Osaka, Japan), ascorbic acid (AA, Wako), and dexamethasone (Sigma-Aldrich) was added 48 h before analysis. Cells were dissolved with 400 mM sodium citrate for 20 min and transferred to microtubes. After centrifugation, the supernatant was discarded. Cell lysates were prepared by adding 0.1% Triton X-100, followed by sonication on ice for 10 min (Bioruptor, Diagenode, Seraing, Belgium) and centrifugation at 12,000 rpm for 15 min at 4 °C. ALPase activity was measured using a LabAssay™ ALP kit (Wako), and total protein concentration was measured using a Pierce™ BCA Protein Assay Kit (Thermo Fisher Scientific Inc.), according to the manufacturers’ instructions. Absorbance was measured at 405 nm and 562 nm, respectively.

### 2.5. Quantitative Reverse Transcription-Polymerase Chain Reaction (qRT-PCR)

To evaluate osteo-odontogenic differentiation, 2 × 10^4^ cells/well were seeded into 24-well microplates containing 300 μL of alginate–gelatin hydrogels. After acrosslinking with the indicated calcium chloride concentration, the culture medium was replaced with osteo-odontogenic induction medium containing DMEM, 5% FBS, β-GP, AA and dexamethasone. The constructs were dissolved with 400 mM sodium citrate for 20 min and centrifuged at 12,000 rpm for 15 min at 4 °C. RNA was extracted and reverse-transcribed into cDNA using M-MLV transcriptase. Quantitative real-time PCR (qRT-PCR) was performed in a 20 µL reaction volume using a LightCycler™ Nano instrument (Roche, Basel, Switzerland). The mRNA expression levels of dentin phosphosialoprotein (DSPP), dentine matrix protein-1 (DMP-1), bone sialoprotein (BSP), osteocalcin (OCN), osteopontin (OPN), and runt-related transcription factor-2 (Runx-2) were quantified. Relative gene expression was calculated using the 2^−ΔΔCt^ method and normalized to β-actin mRNA expression. Quantitative real-time RT-PCR primer sequences and reaction conditions are listed in Table 1. Primers were obtained from Invitrogen.

### 2.6. Mineralization Nodule of Alizarin Red Staining

Mineralized nodules on the hydrogel surface were evaluated after 5 and 7 days of culture. Cells were seeded at 4 × 10^4^ cells/well with 400 μL of alginate gelatin hydrogels into 12-well microplates. Osteo-odontogenic induction medium contained DMEM, 5% FBS, β-GP, AA, and dexamethasone was added 48 h before analysis. The constructs were fixed in 10% neutral-buffered formalin (Wako) for 10 min and stained with 0.5% Alizarin Red S (Wako) at pH 4.2 for 20 min. Mineralized nodules were visualized using an inverted microscope (Nikon ECLIPSE Ti 2 series). The number of stained nodules was quantified from representative images taken from 4 to 5 randomly selected fields per sample, using ImageJ software (version 1.53, USA).

### 2.7. Statistical Analysis

All experiments were performed in triplicate, and the results are presented as mean ± standard deviation. Statistical comparisons among groups were performed using one-way analysis of variance (ANOVA) followed by Tukey’s HSD post hoc test with Social Science Statistics web-based tool. A *p*-value < 0.05 was considered statistically significant.

## 3. Results

### 3.1. Cell Proliferation Response

**Figure 2:**
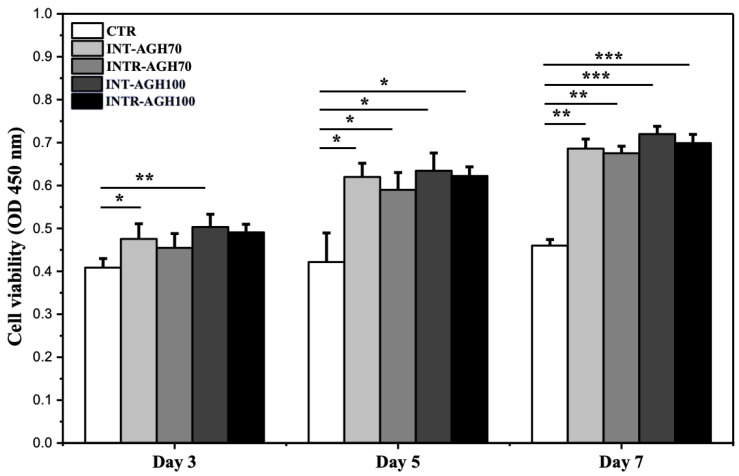
Proliferation of MDPC-23 cells cultured in INT-AGH and INTR-AGH constructs prepared with different calcium cholaride concentrations, compared with the control group (OD450; * *p* < 0.05, ** *p* < 0.01, and *** *p* < 0.001).

The proliferation of MDPC-23 cells was evaluated on days 3, 5, and 7, and the results are presented in Figure 2. The INT-AGHs group exhibited significantly higher cell viability compared to the control group during the observation period (*p* < 0.05). Although no statistically significant difference was detected between the 70 mM and 100 mM CaCl_2_ groups, the 100 mM condition consistently showed slightly higher values. By day 7, the INT-AGH100 and INTR-AGH100 groups showed approximately 30% and 20% greater proliferation, respectively, than the control group. Because the 100 mM condition showed consistently higher values across assays, it was selected for subsequent analyses.

### 3.2. Cell Morphology and Cytoskeleton Visualization

**Figure 3:**
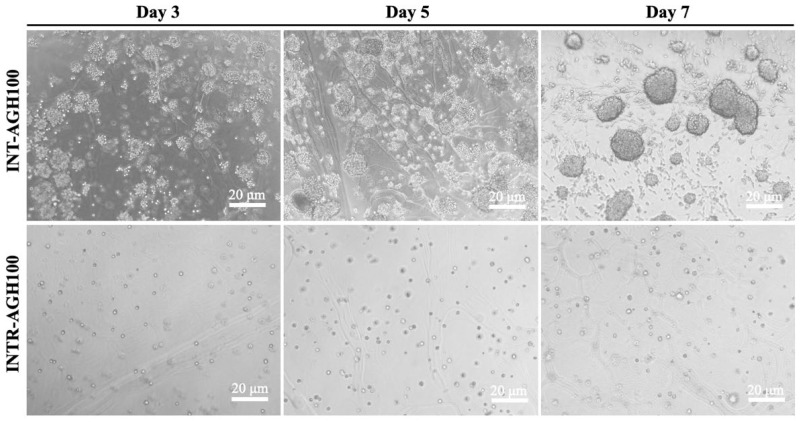
Representative phase-contrast images of cell morphology in the INT-AGH100 and INTR-AGH100 groups on days 3, 5, and 7.

**Figure 4:**
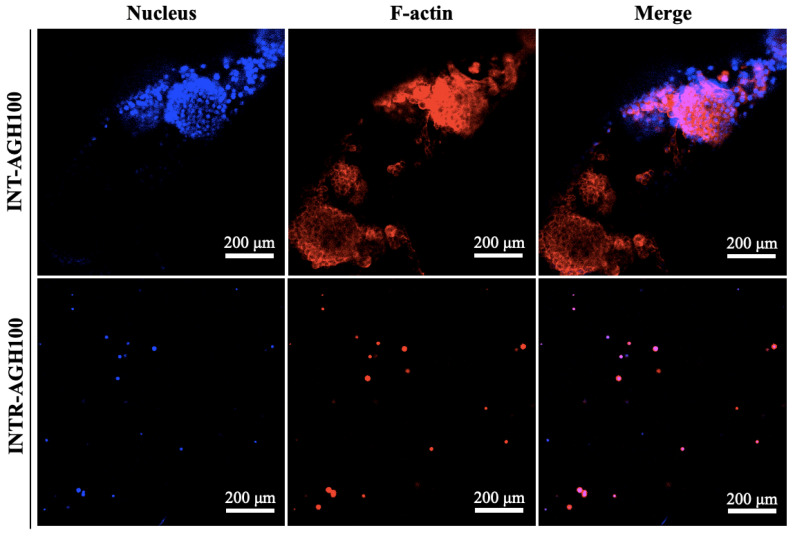
Representative fluorescence images of cytoskeletal morphology in the INT-AGH100 and INTR-AGH100 groups after 7 days of culture.

Microscopic observation showed that cells proliferated successfully in both alginate–gelatin hydrogel constructs. The INT-AGH100 group exhibited larger cell diameters and more diverse characteristics compared to the INTR-AGH100 group (Figure 3). However, distinct morphological differences were observed between the two groups. In the INTR-AGH100 group, cells predominantly retained a rounded morphology within the hydrogel matrix. In contrast, the INT-AGH100 constructs displayed irregular cell clusters on the hydrogel surface, with prominent actin fibers approximately 1–4 μm in diameter after 7 days of culture (Figure 4). Cells remained viable in both groups, and the apparent spreading area was greater in the INT-AGH100 group than in the INTR-AGH100 group throughout the observation period.

### 3.3. Alkaline Phosphatase Activity

**Figure 5:**
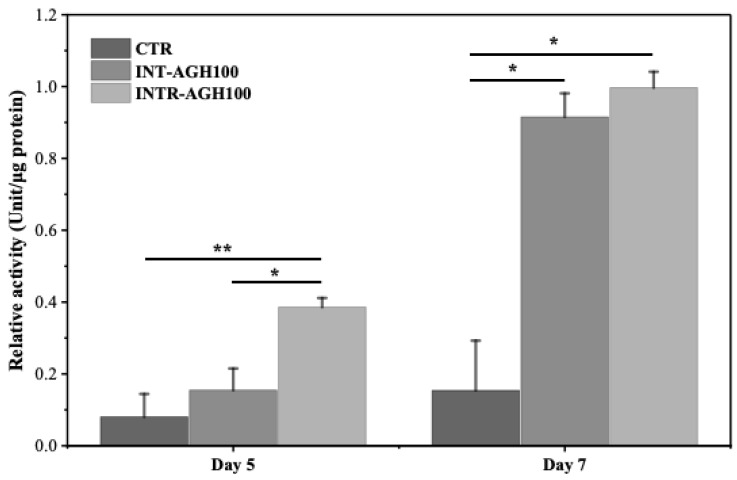
Quantitative analysis of ALPase activity on days 5 and 7 (* *p* < 0.05 and ** *p* < 0.01).

ALPase activity was higher in the hydrogel groups than in the control group and was significantly elevated in the INTR-AGH100 group on days 5 and 7 (* *p* < 0.05). These findings indicate that the 100 mM calcium chloride condition supported enhanced early osteo-odontogenic activity. However, no statistically significant difference in ALPase activity was observed between the INT-AGH100 and INTR-AGH100 groups (*p* > 0.05) as shown in Figure 5. The ALPase level in the INT-AGH100 group at day 5 remained close to that of the control group.

### 3.4. Osteo-Odontogenesis Differentiation

**Figure 6:**
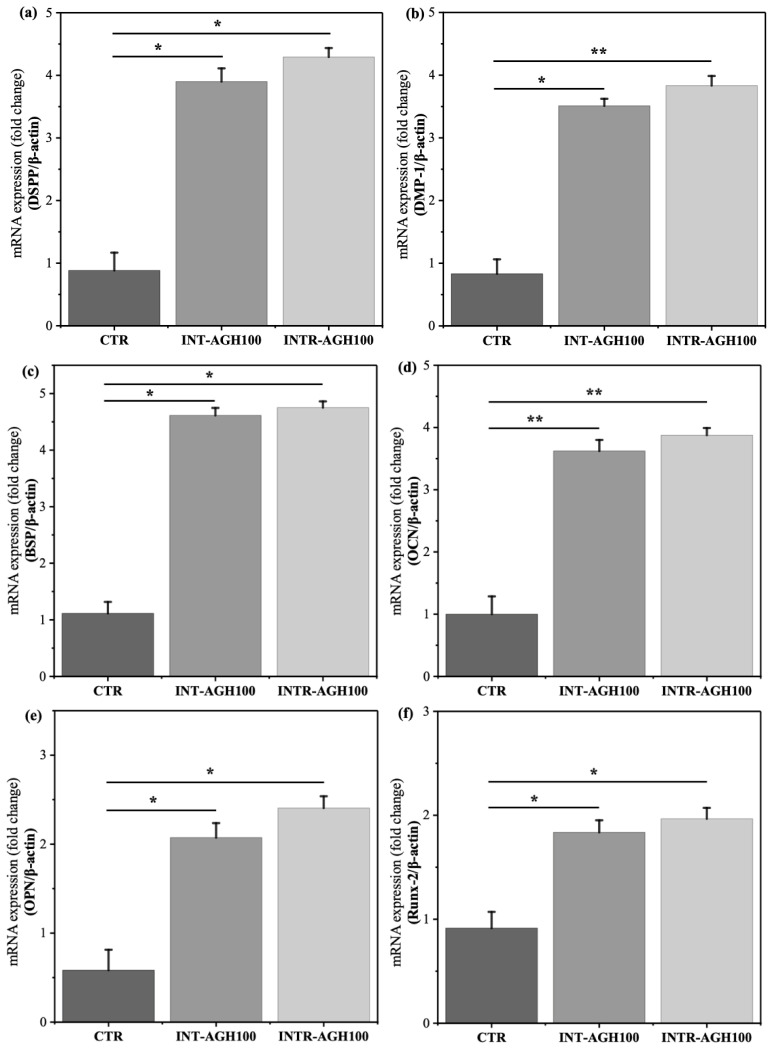
Expression of osteo-odontogenic differentiation markers: (**a**) DSPP, (**b**) DMP-1, (**c**) BSP, (**d**) OCN, (**e**) OPN, and (**f**) RUNX-2 (* *p* < 0.05, ** *p* < 0.01).

Consistent with the ALPase results, qRT-PCR analysis revealed increased expression of osteo-odontogenic markers in the hydrogel groups. After 7 days of culture, the mRNA expression levels of DSPP, DMP-1, BSP, OCN, OPN, and Runx-2 were significantly higher in the AGH groups than in the control group with * *p* < 0.05 (Figure 6). In particular, BSP and DSPP showed marked upregulation, with approximately 4.7-fold and 4.2-fold increases, respectively. While, the expression results between INT-AGH100 and INTR-AGH100 demonstrated not significant differences (*p* > 0.05). However, these findings support the capacity of the alginate–gelatin hydrogel system to promote osteo-odontogenic differentiation in odontoblast-like cells.

### 3.5. Mineralization Nodule Formation

**Figure 7:**
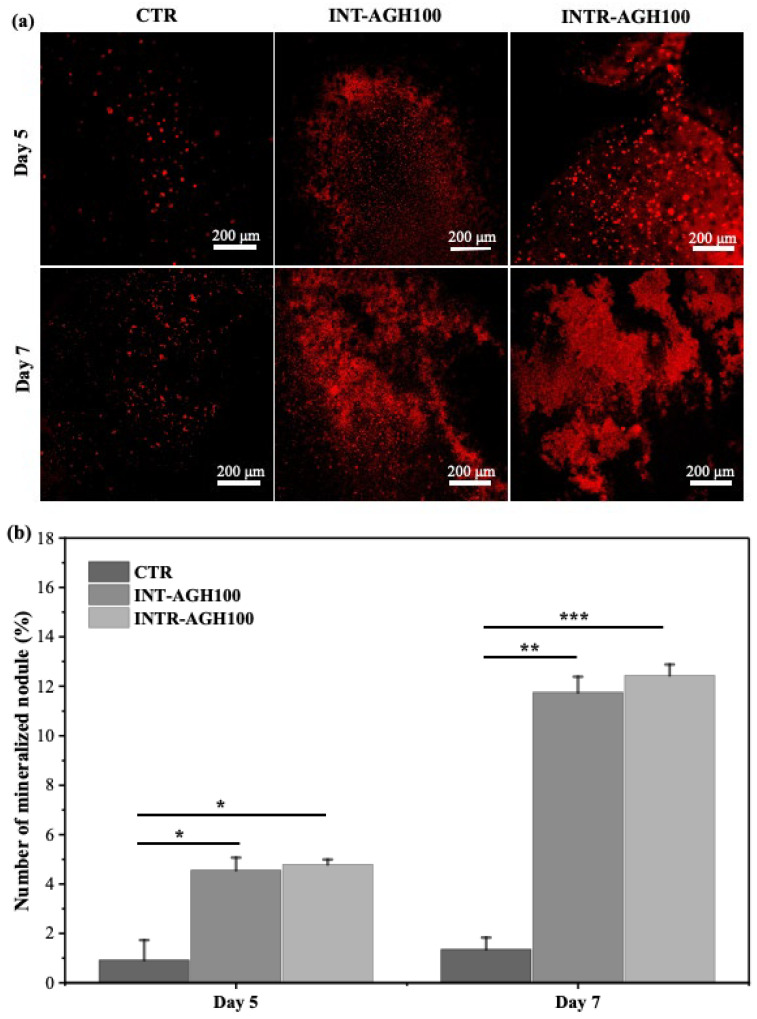
Mineralization nodule formation in INT-AGH100 and INTR-AGH100 compared with the control group after 5 and 7 days of culture: (**a**) representative microscopic images and (**b**) quantitative analysis of mineralized nodules. (* *p* < 0.05, ** *p* < 0.01, and *** *p* < 0.001).

To evaluate late-stage mineralization, Alizarin red S staining was performed. Both INTR-AGH100 and INT-AGH100 groups showed more abundant mineralized nodules than control group during the study period (Figure 7a). Quantitative analysis showed that mineralized nodule formation in the hydrogel groups significantly increased further on day 7 compared with day 5 in both groups (* *p* < 0.05), indicating progressive mineralization over time. Furthermore, the statistical results were confirmed not significant different between INT-AGH100 and INTR-AGH100 group (*p* > 0.05) (Figure 7b).

## 4. Discussion

The successful development of dentin regeneration therapy depends on the establishment of scaffold systems capable of supporting cell survival, proliferation, differentiation, and mineralized matrix formation within a controlled microenvironment [3,8]. In this context, scaffold-based approaches are expected not only to provide structural support but also to create a biologically permissive environment for cellular activity related to dentin repair and regeneration [4,13]. In the present study, calcium chloride–crosslinked alginate–gelatin hydrogels were evaluated as three-dimensional carriers for odontoblast-like cells. Within the limitations of the present experimental design, the findings suggest that hydrogels crosslinked with 100 mM CaCl_2_ may provide a favorable environment for cell proliferation, early osteo-odontogenic activity, and mineralization [2,9]. The increase in cell proliferation observed at higher CaCl_2_ concentrations may be related, at least in part, to changes in hydrogel architecture induced by ionic crosslinking [20]. Previous studies have suggested that calcium chloride–crosslinked alginate–gelatin hydrogels can form porous networks that facilitate cell retention and matrix interaction [22,31]. In the present study, preliminary observations indicated that CaCl_2_ concentrations below 50 mM did not produce stable gelation, whereas concentrations above 50 mM allowed reproducible hydrogel formation [27]. Although the difference between 70 mM and 100 mM was not statistically significant in the proliferation assay, the 100 mM condition consistently yielded higher values and was therefore selected for further evaluation [21,22]. This interpretation should nevertheless be made with caution, because the present findings indicate a concentration-dependent trend rather than a definitively established dose–response relationship [17,20]. In addition, mechanical characterization of the hydrogels was not performed, even though scaffold stiffness, viscoelasticity, and network density may influence cell behavior. Future studies will therefore be required to clarify how the mechanical properties of calcium chloride–crosslinked alginate–gelatin hydrogels relate to odontoblast differentiation-inducing capacity and to determine the optimal crosslinking condition more precisely.

The two seeding strategies produced distinct morphological outcomes. In the inter-hydrogel model, cells were located on the hydrogel surface and exhibited greater spreading and cluster formation, which is consistent with greater exposure to surface cues and two-dimensional attachment behavior [27,32]. In contrast, cells in the intra-hydrogel model remained rounded within the hydrogel matrix, reflecting confinement within a three-dimensional environment [31]. These differences may be biologically relevant, because cell morphology and cell–matrix interactions are closely associated with lineage commitment and differentiation behavior in hydrogel-based cultures [27,33]. However, because the present study did not include quantitative morphometric analysis, these observations should be interpreted as descriptive rather than definitive [21,22]. The relatively high ALPase activity observed in the INTR-AGH100 group may suggest that the encapsulated three-dimensional environment supports early differentiation-related activity more effectively than surface seeding alone, although this interpretation requires further confirmation.

The gene expression findings also support the osteo-odontogenic potential of the hydrogel system [18,33]. DSPP and DMP-1 are well-established markers associated with odontogenic differentiation and dentin matrix formation [33,34], and their upregulation in the hydrogel groups suggests that the microenvironment created by the calcium chloride–crosslinked alginate–gelatin hydrogels may favor differentiation toward an odontoblast-like phenotype [33]. The increased expression of BSP and OCN may further indicate active mineralization-related signaling and progression toward matrix maturation [16,18]. By contrast, the expression patterns of OPN and Runx-2 should be interpreted carefully, because these markers may vary according to the stage of differentiation and the relative contribution of osteogenic and odontogenic pathways [35]. Accordingly, the present results do not support strong mechanistic conclusions regarding lineage regulation, but they do suggest an overall shift toward enhanced osteo-odontogenic differentiation in the hydrogel groups.

The mineralization results obtained by Alizarin red S staining were generally consistent with the molecular findings. Mineralized nodules were more abundant in the AGH groups than in the control group, and the INTR-AGH100 group showed sustained mineral deposition during the experimental period. These observations may indicate that the encapsulated three-dimensional environment is favorable for continued mineralized matrix formation [27]. Previous studies have likewise reported that alginate–gelatin hydrogel systems can enhance mineralized matrix formation and differentiation-related marker expression in dental pulp stem cell models [7,16,36]. Nevertheless, the present findings should be interpreted within the limits of an in vitro cell-line model, and they should not be taken as direct evidence of dentin regeneration in vivo.

From the perspective of dentin regeneration therapy, the present hydrogel system may be viewed as a potentially useful experimental scaffold platform [17]. A clinically relevant dentin-regenerative scaffold would ideally combine biocompatibility, reproducible gelation, support for pulp-derived or odontogenic cells, and the ability to promote organized mineralized tissue formation within the confined dentin-pulp state [33]. The present results suggest that calcium chloride-crosslinked alginate–gelatin hydrogels may fulfill some of these requirements at the in vitro level. However, the transition from an experimental hydrogel model to a clinically applicable regenerative material requires substantially broader validation, including analyses of mechanical properties, degradation behavior, ion-release kinetics, tissue integration, and biological performance in vivo [27,32]. Thus, although the present study provides useful preliminary information for scaffold design, its translational implications remain limited at this stage.

Several limitations of the present study should therefore be acknowledged. First, only a limited range of calcium chloride concentrations was examined, and a broader concentration series would be necessary to define the optimal crosslinking condition more precisely [35]. Second, the mechanical characterization of the hydrogels such as rheological properties and FT-IR analysis were not characterized, although these properties may influence proliferation and differentiation [27]. Third, calcium (Ca^2+^) release from the hydrogels into the culture medium was not quantified, even though it may contribute to the observed biological responses [2,37]. Fourth, a conventional two-dimensional culture control without hydrogels was not included, because the primary aim of the study was to compare different calcium chloride–crosslinked hydrogel conditions rather than hydrogel culture with standard tissue-culture plastic [18,36]. Finally, the study was conducted exclusively in vitro using a single odontoblast-like cell line. Accordingly, further studies involving broader material characterization, quantitative morphological analysis, mechanistic investigation, and in vivo validation will be essential before the present findings can be extended to a therapeutic framework for dentin regeneration.

Taken together, the present results suggest that calcium chloride–crosslinked alginate–gelatin hydrogels, particularly under the 100 mM condition, can provide a biologically favorable three-dimensional microenvironment for odontoblast-like cells under the conditions tested. While the current evidence remains preliminary, the observed increases in proliferation, differentiation-associated marker expression, and mineralized nodule formation indicate that this hydrogel system may serve as a useful in vitro model for further investigation of scaffold-guided dentin regeneration.

## 5. Conclusions

Within the limitations of this in vitro study, alginate–gelatin hydrogels crosslinked with 100 mM calcium chloride supported increased proliferation, osteo-odontogenic marker expression, and mineralized nodule formation in odontoblast-like cells. Although no significant differences were observed between the inter-hydrogel and intra-hydrogel groups in all assays, the two seeding strategies produced distinct morphological patterns, and the intra-hydrogel condition showed relatively high early differentiation-related activity. These findings suggest that calcium chloride-crosslinked alginate–gelatin hydrogels may provide a supportive three-dimensional microenvironment for odontoblast-like cells and may serve as a useful in vitro scaffold model for further studies of dentin-related tissue regeneration.

## Figures and Tables

**Table 1:** Oligonucleotide primer sequences used for quantitative real-time PCR analysis of expression-markers.

Target Gene	Primer Sequence (5′–3′)		Product Size (bp)
	Forward	Reverse	
DSPP	TCAATGGCGGGTGCTTTAGA	TGCTCACTGCACAACATGAAGA	111
DMP-1	CGTTCCTCTGGGGGCTGTCC	CCGGGATCATCGCTCTGCATC	577
BSP	CTGCTTTAATCTTGCTCTG	CCATCTCCATTTTCTTCC	211
OCN	AGCTCAACCCCAATTGTGAC	AGCTGTGCCGTCCATACTTT	190
OPN	TTTCCCTGTTTCTGATGAACAGTAT	CTCTGCTTATACTCCTTGGACTGCT	228
Runx-2	CCACAGAGCTATTAAAGTGACAGTG	AACAAACTAGGTTTAGAGTCATCAAGC	87
β-actin	AACCCTAAGGCCAACAGTGAAAAG	TCATGAGGTAGTCTGTGAGGT	240

## Data Availability

Data is contained within the article.
